# Impact of purulent complications and sepsis on cardiovascular system in patients with type 2 diabetes

**DOI:** 10.1186/cc14009

**Published:** 2014-12-03

**Authors:** E Shalaeva, B Babadjanov, U Pulatov, N Dadabayeva, A Shalaeva

**Affiliations:** 1 Republican Center of Purulent Surgery and Complications of Diabetes, Tashkent Medical Academy, Tashkent, Uzbekistan

## Introduction

Purulent complications in patients with type 2 diabetes are usually severe, often complicated by sepsis and require emergency surgery. Noncardiac surgery is associated with a 7 to 11% complication rate and mortality of 0.8 to 1.5% [1], up to 42% are cardiac reasons [2]. After surgery, 2% of patients suffer major cardiac complications [3], and 8% show evidence of significant myocardial injury [2]. The aim of this study was to identify the impact of purulent complications and sepsis on cardiovascular system in patients with type 2 diabetes.

## Methods

We analyzed 112 consecutive patients (54 men and 58 women) aged 57.2 ± 8.4 years with purulent-necrotic complications (gangrene, phlegmon, and abscess) of type 2 diabetes and sepsis in 2013. We compared laboratory and instrumental data (blood tests, ECG, echocardiography and others), which were previously obtained in the same patients receiving inpatient treatment before sepsis (2011 to 2012).

## Results

Gangrene of lower extremities in 59 (52.7%) prevailed among purulent complications. After the development of sepsis we detected in all patients significantly increased heart rate, respiratory rate per minute, leukocytosis, anemia, worse glucose metabolism and renal function (Table 1). Congestive heart failure became more severe. This was confirmed by decrease of left ventricle ejection fraction (55.2 ± 5.1% before sepsis vs. 49.3 ± 4.1% after) and increase brain natriuretic peptide (291.4 ± 34.5 ng/ml vs. 395.2 ± 28.1 ng/ml, *P *< 0.001). Prior sepsis in 66 (58.9%) of patients with arterial hypertension was observed, after in 88 (78.6%). After admission to the centre, patients had no signs of septic shock. In 13 (11.6%) patients, the perioperative period was complicated by acute myocardial infarction, which was accompanied by a fall in blood pressure. We detected an increase of the functional class of stable angina, congestive heart failure, 4.2 times increased incidence of unstable angina, 2.6 times ventricular and four times supraventricular extra systole after septic complications (Table 2).

**Table 1:** Hemodynamic parameters and blood tests in patients with purulent complications of type 2 diabetes and sepsis

Parameter	Before sepsis (*n *= 112)	After sepsis (*n *= 112)	*P *value
Heart rate (beats/minute)	78.4 ± 15.2	112.5 ± 18.9	< 0.001
Respiratory rate (breaths/minute)	18.0 ± 2.0	29.5 ± 5.5	< 0.001
Systolic BP (mmHg)	155.7 ± 35.4	154.2 ± 58.5	n.s.
Diastolic BP (mmHg)	90.4 ± 10.3	91.9 ± 8.6	n.s.
**Left ventricle ejection fraction (%)**	**55.2 ± 5.1**	**49.3 ± 4.1**	**0.033**
Fasting plasma glucose (mmol/l)	8.4 ± 2.5	15.4 ± 4.8	< 0.001
Two-hour plasma glucose (mmol/l)	10.2 ± 2,8	19.9 ± 3.3	< 0.001
**HbA1c (%**)	**8.4 ± 0.5**	**12.1 ± 0.5 **	**< 0.001**
Hemoglobin (g/l)	121.5 ± 12.5	105.4 ± 11.7	0.04
White count (10^3^)	6.7 ± 1.2	14.4 ± 2.1	< 0.001
Fibrinogen (mg%)	411.6 ± 103.6	715.4 ± 215.5	< 0.001
Blood urea (mmol/l)	6.1 ± 2.9	8.8 ± 2.5	0.011
Blood creatinine (mmol/l)	88.4 ± 18.5	105.6 ± 17.3	0.02
Brain natriuretic peptide (ng/ml)	291.4 ± 34.5	395.2 ± 28.1	< 0.001

BP, blood pressure; HbA1c, glycosylated hemoglobin A1c.

**Table 2:** Cardiovascular comorbidity in patients with type 2 diabetes before and after purulent-necrotic complications and sepsis

Parameter	Before sepsis (*n *= 112)	After sepsis (*n *= 112)
**Insulin dependence**	**42 (37.5)**	**112 (100)**
Normal blood pressure (110 to 139 mmHg)	46 (41.1)	11 (9.8)
**Arterial hypertension**	**66 (58.9)**	**88 (78.6)**
First degree (140 to 159 mmHg)	33 (29.5)	21 (8.9)
Second degree (160 to 179 mmHg)	21 (18.6)	43 (38.4)
Third degree (>180 mmHg)	12 (10.7)	24 (21.4)
Arterial hypotension (<90 mmHg)	-	13 (11.6)
CAD, stable angina	108 (94.6)	82 (73.2)
FC I	18 (16.1)	-
FC II	29 (25.9)	18 (16.1)
FC III	52 (46.4)	45 (40.2)
FC IV	9 (8.0)	19 (17.0)
**CAD, unstable angina**	**4 (3.6)**	**17 (15.2)**
**Acute myocardial infarction**	-	**13 (11.6)**
Postinfarction cardiosclerosis	7 (6.3)	7 (6.3)
Atrial fibrillation	7 (6.3)	7 (6.3)
Supraventricular arrhythmia	3 (2.7)	12 (10.7)
Ventricular arrhythmia	14 (12.5)	36 (32.1)
Congestive heart failure	112 (100)	112 (100)
FC II (NYHA)	76 (67.8)	26 (23.2)
FC III (NYHA)	36 (32.1)	65 (58)
FC IV (NYHA)	-	21 (18.7)
Abscesses of the lower extremity	-	22 (19.6)
Phlegmon of the lower extremity	-	31 (27.7)
Gangrene of lower extremity	-	59 (52.7)

Data presented as *n *(%). CAD, coronary artery disease; FC, functional class; NYHA, New York Heart Association.

## Conclusion

After the development of purulent complications and sepsis in patients with type 2 diabetes, we observed increased incidence of arterial hypertension, arrhythmias, worsened severity of coronary artery disease and congestive heart failure. Perioperative risk of acute myocardial infarction amounted to 11.6%.
